# Supplementary material for: Leveraging a hybrid convolutional gated recursive diabetes prediction and severity grading model through a mobile app
Source: PeerJ Comput Sci. 2025 Feb 12;11:e2642. doi: 10.7717/peerj-cs.2642 (PMC11888868; doi:10.7717/peerj-cs.2642)

**Technical changes**

[**#**](https://peerj.com/manuscripts/103103/validate/#ai-applications)**AI Applications**

**Please edit your manuscript and/or supplemental files to include the following:**

- **Reproducibility**
  - **README file**

**NOTE: The README file should introduce and explain your code, as well as provide steps for implementation.**

Response: The Reproducibility statement is attached in the manuscript in conclusion part and highlighted in green color. It is mentioned as “The proposed model will consistently produce the improved outcome over diverse datasets to be implemented in a clinical practice, ensuring its reproducibility.”

Moreover, the code explanation and steps for implementation is attached below.

**CODE EXPLANATION AND STEPS FOR IMPLEMENTATION:**

1. Initially the required libraries are imported and the dataset is read as .csv file


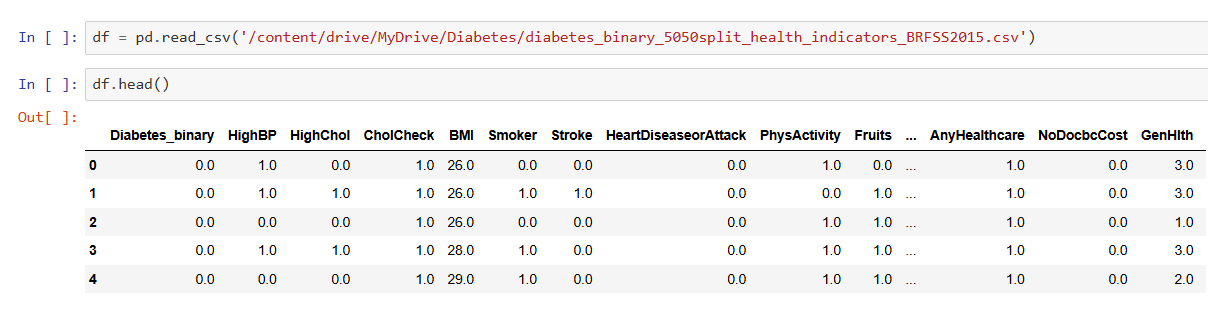


1. Next, the pre-processing steps like missing data imputation, irrelevant feature removal steps are applied and the new dataset is built


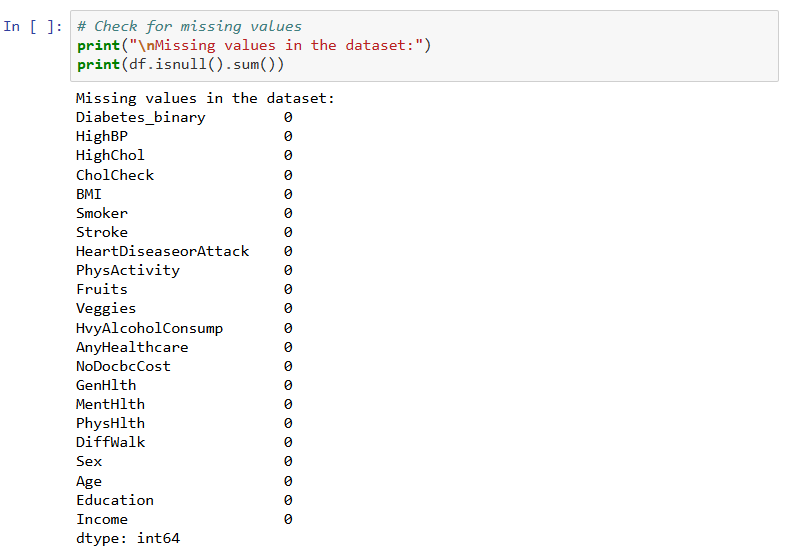


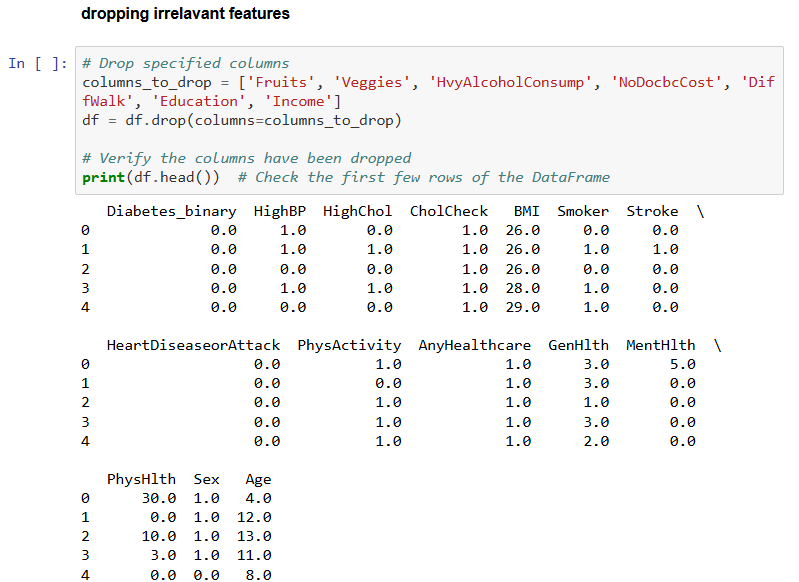


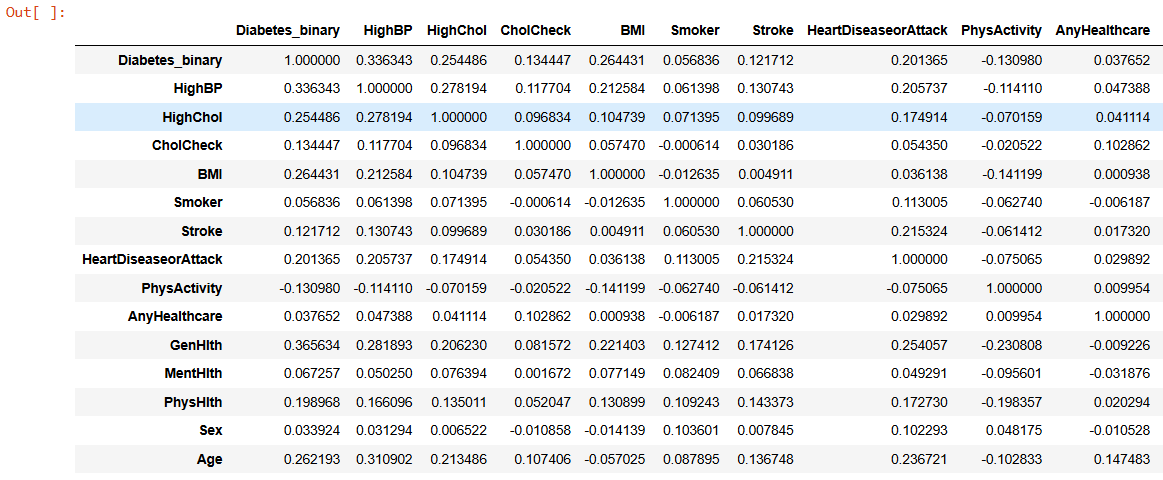


1. In the next step, the data visualization is done to explore the dataset and potentially generate hypotheses about the relationships between BMI, physical health, and diabetes.


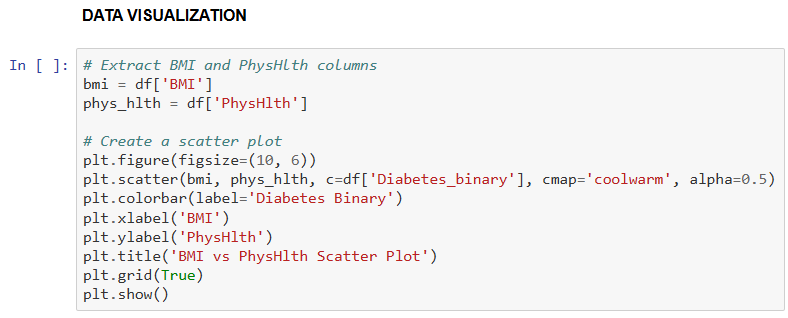


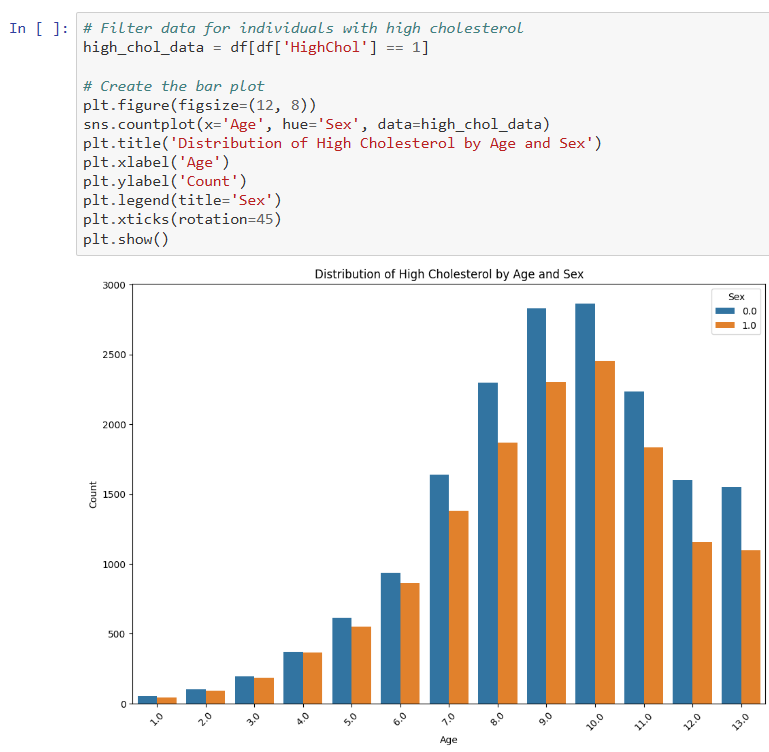


This indicates the distribution of cholesterol level among different age groups and among male and female. The higher bars for older age groups indicate that high cholesterol is more prevalent among older individuals. The male bars consistently higher across age groups indicate that males have a higher incidence of high cholesterol compared to females in this dataset. This may help to inform decisions, develop hypotheses for further analysis, or guide interventions aimed at reducing high cholesterol in specific demographic groups


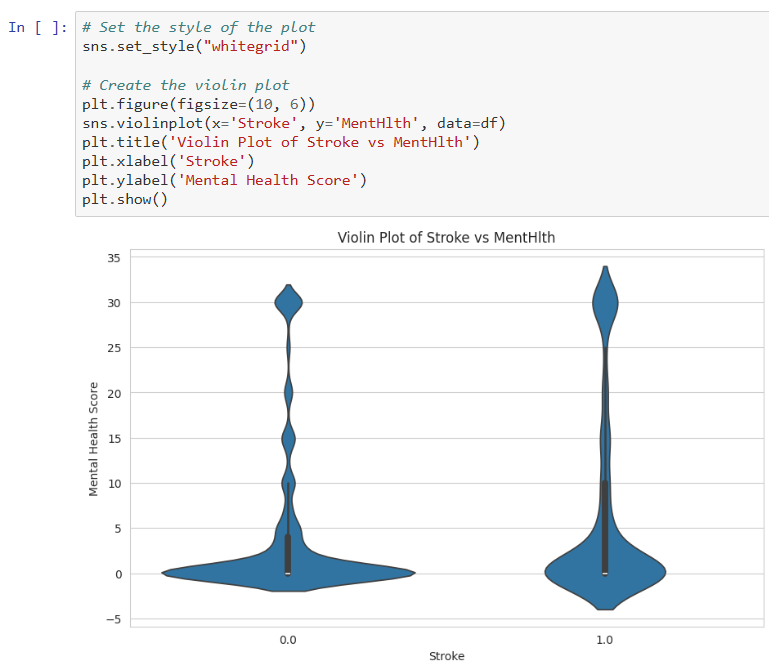


The violin plot shows the distribution of mental health scores for individuals with and without a history of stroke. The width of the violin at different y-values indicates the density of data points at that score level. By comparing the shapes and spread of the violins for the two categories (Stroke vs. No Stroke), the differences in mental health scores between the groups can be noted. This plot helps to identify whether there's a noticeable difference in mental health scores between individuals who have had a stroke and those who haven't.

1. In the next step, the correlation matrix is calculated to identify which variables have strong correlations, which might be useful for further analysis or feature selection. The highly correlated variables can be used for feature engineering or removing one of them to reduce redundancy.


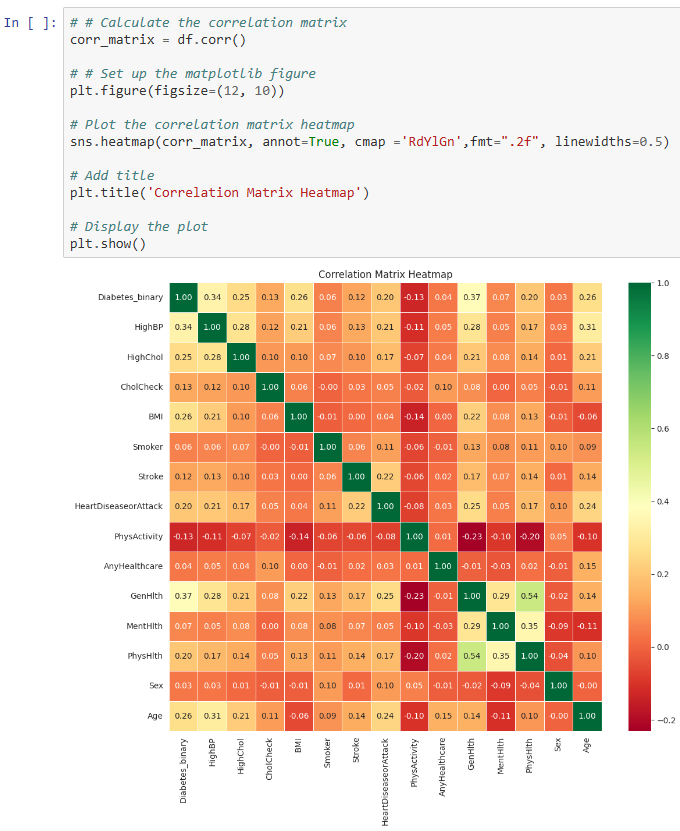


1. Then, Moving Average smoothing is done to smooth out short-term fluctuations and highlight longer-term trends in data. It calculates the average of a set number of consecutive data points (the window size). In this case, the window size is 3, meaning each value in the smoothed DataFrame is the average of itself and the two preceding values.


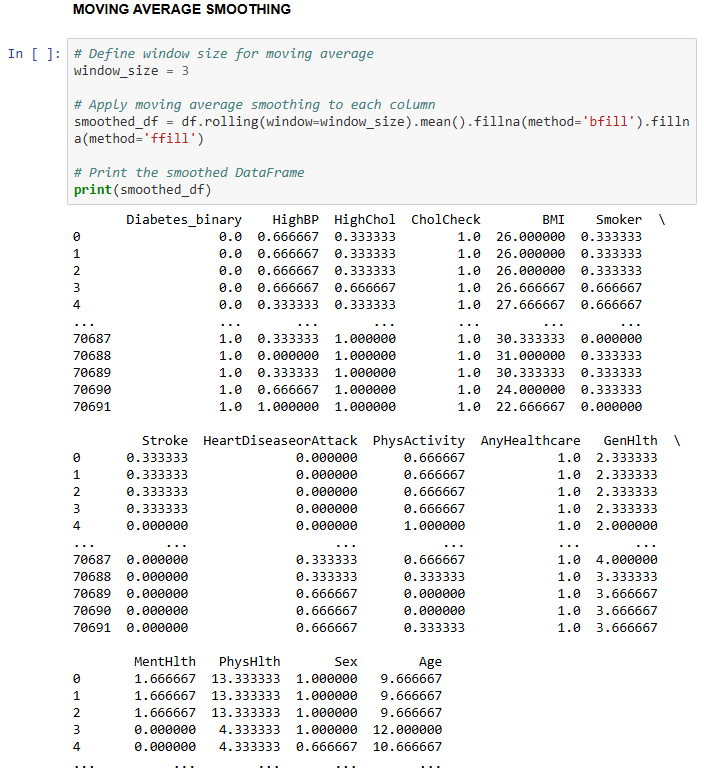


1. The dataset is divided into training and testing sets (70% training, 30% testing) ensuring the even distribution of data for both training and testing sets.
2. Hybridizing CNN and GRU model is the next step.
3. In CNN model, Conv1D Layer extracts features from sequences, MaxPooling1D Layer reduces dimensionality, flatten Layer converts 2D data to 1D for dense layers, Dense Layer is a final layer with 64 units for feature learning. Moreover, in GRU model, GRU Layer captures sequential patterns, Dense Layer has 64 units for additional feature learning. Altogether, this Unified Model combines CNN Features includes convolutional layers and pooling layer; Additional Dense Layers for further feature processing before the final output; Output Layer uses a sigmoid activation function for binary classification. For compilation, the Adam Optimizer with a learning rate of 0.001. Binary cross-entropy Loss Function is chosen for binary classification.
4. Now the model is trained


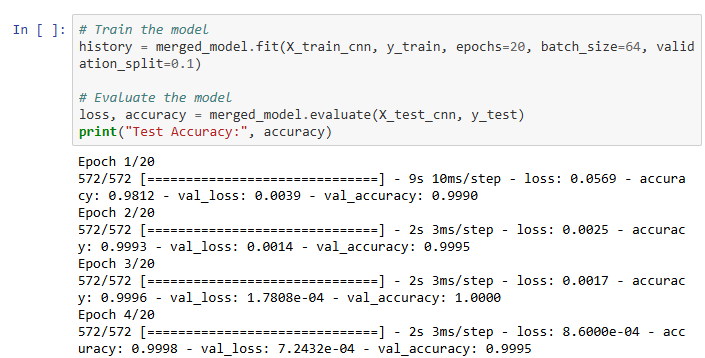


1. The training and validation accuracy and loss graph is plotted


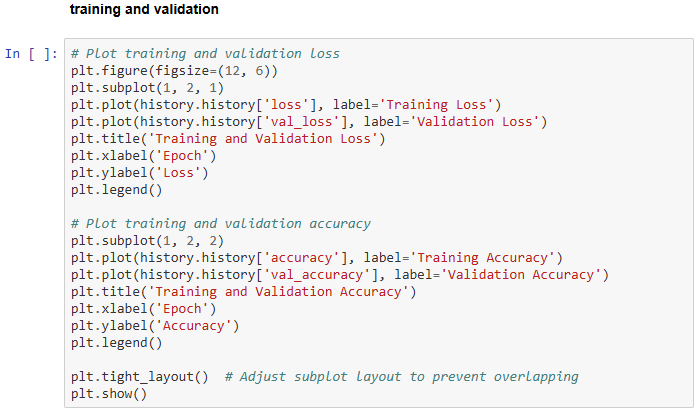


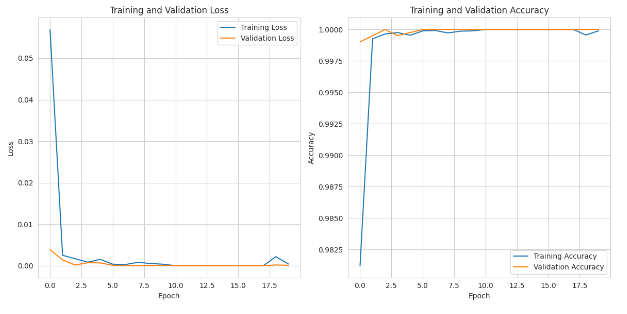


Training Loss shows how the model's loss decreased over epochs during training. Validation Loss indicates how well the model generalizes to unseen data. A decrease in both training and validation loss suggests good model performance and learning. Training Accuracy shows the accuracy of the model on the training data. Validation Accuracy shows the accuracy on the validation set, reflecting the model's ability to generalize. An increasing trend in both training and validation accuracy indicates that the model is learning effectively.

1. Now the confusion matrix is plotted for binary classification of diabetes. The confusion matrix for diabetes classification reveals impressive performance. With 8202 true negatives (instances correctly identified as not having diabetes) and 9207 true positives (instances correctly identified as having diabetes), the model accurately identifies both non-diabetic and diabetic cases. Remarkably, it generates almost no false positives (instances incorrectly labeled as having diabetes as no diabetes), the single false negative indicates a minor misclassification (instances incorrectly identified as not having diabetes as diabetes).


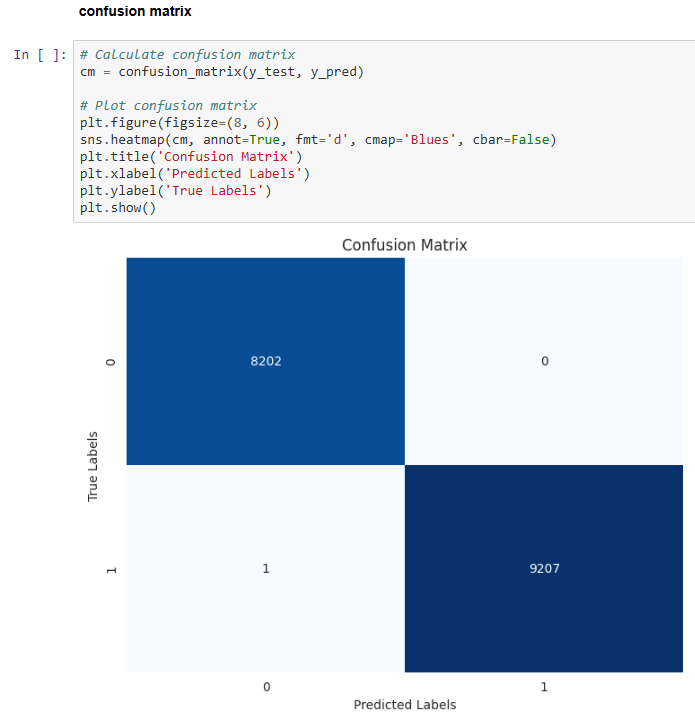


1. Next, the ROC Curve is plotted. The AUC score provides a single number summary of the model's overall performance. The higher AUC indicates the better the model is at distinguishing between the positive and negative classes.
2. Now the model is saved and the data that are identified as diabetes is taken separately for severity level classification.


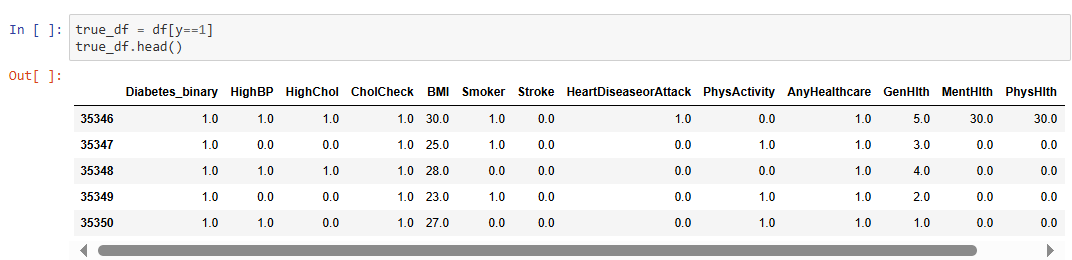


1. Now clustering anlaysis is done to group the data into 3 classes as low, Moderate and high. KMeans is used to assign data points to clusters, which are then mapped to severity levels.


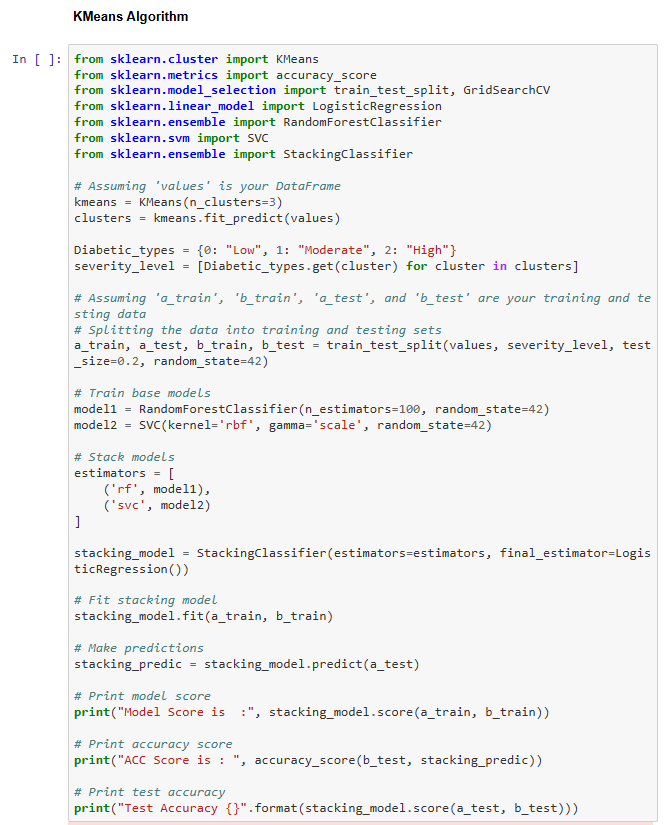


1. The clustering algorithm groups the data into 3 classes as shown below


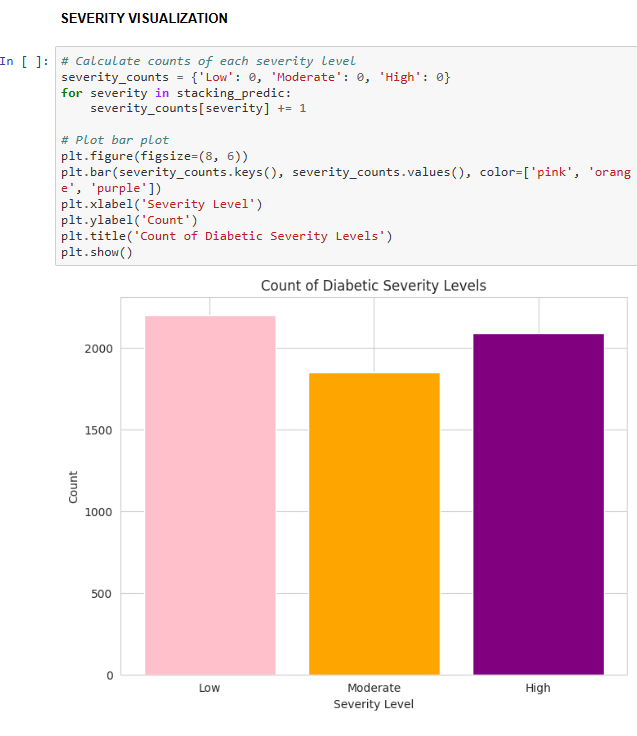


1. In the next step, the confusion matrix is built for severity level classification


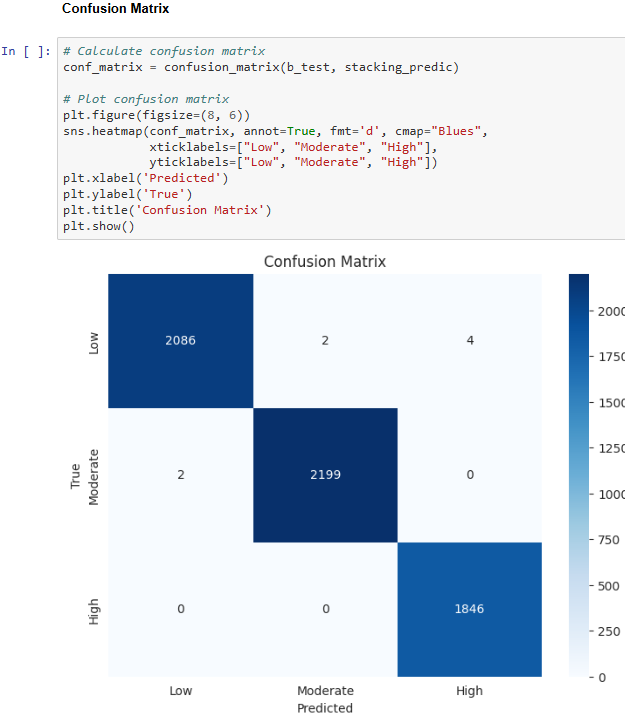


This matrix represents the performance of an algorithm across three categories: Low, Moderate, and High. **Low:** Correctly predicted 2086 instances, **Moderate:** Accurate predictions for 2199 cases, and **High:** Successfully identified 1846 instances. Notably, the model rarely misclassifies, with only a few errors between adjacent categories. Overall, it demonstrates strong performance.

1. The final classification report is then displayed. It indicates the model’s optimal performance in terms of parameters like, accuracy, precision, recall and f1-score.


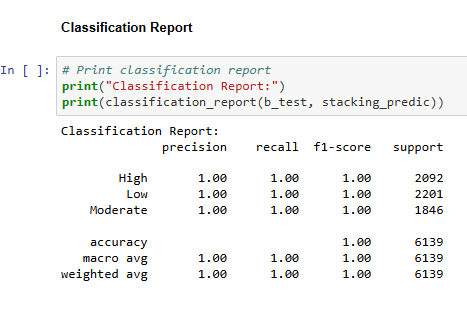

Supplement: Supplemental Information 2 [file peerj-cs-11-2642-s002.docx]
